# Factor Structure Underlying Components of Allostatic Load

**DOI:** 10.1371/journal.pone.0047246

**Published:** 2012-10-24

**Authors:** Jeanne M. McCaffery, Anna L. Marsland, Kelley Strohacker, Matthew F. Muldoon, Stephen B. Manuck

**Affiliations:** 1 Department of Psychiatry and Human Behavior, The Miriam Hospital and Warren Alpert School of Medicine at Brown University, Providence, Rhode Island, United States of America; 2 Department of Psychology, University of Pittsburgh, Pittsburgh, Pennsylvania, United States of America; 3 Heart and Vascular Institute, University of Pittsburgh School of Medicine, Pittsburgh, Pennsylvania, United States of America; 4 Department of Psychology, University of Pittsburgh, Pittsburgh, Pennsylvania, United States of America; Governmental Technical Research Centre of Finland, Finland

## Abstract

Allostatic load is a commonly used metric of health risk based on the hypothesis that recurrent exposure to environmental demands (e.g., stress) engenders a progressive dysregulation of multiple physiological systems. Prominent indicators of response to environmental challenges, such as stress-related hormones, sympatho-vagal balance, or inflammatory cytokines, comprise primary allostatic mediators. Secondary mediators reflect ensuing biological alterations that accumulate over time and confer risk for clinical disease but overlap substantially with a second metric of health risk, the metabolic syndrome. Whether allostatic load mediators covary and thus warrant treatment as a unitary construct remains to be established and, in particular, the relation of allostatic load parameters to the metabolic syndrome requires elucidation. Here, we employ confirmatory factor analysis to test: 1) whether a single common factor underlies variation in physiological systems associated with allostatic load; and 2) whether allostatic load parameters continue to load on a single common factor if a second factor representing the metabolic syndrome is also modeled. Participants were 645 adults from Allegheny County, PA (30–54 years old, 82% non-Hispanic white, 52% female) who were free of confounding medications. Model fitting supported a single, second-order factor underlying variance in the allostatic load components available in this study (metabolic, inflammatory and vagal measures). Further, this common factor reflecting covariation among allostatic load components persisted when a latent factor representing metabolic syndrome facets was conjointly modeled. Overall, this study provides novel evidence that the modeled allostatic load components do share common variance as hypothesized. Moreover, the common variance suggests the existence of statistical coherence above and beyond that attributable to the metabolic syndrome.

## Introduction

Measured levels of seemingly disparate physiological processes tend to covary in populations and to aggregate within individuals. Two widely-used, but interrelated, metrics have been proposed to express shared physiologic variance in the context of health risk. The first is called the metabolic syndrome and figures prominently in cardiovascular disease and diabetes risk epidemiology. The metabolic syndrome captures the co-occurrence of several cardiometabolic abnormalities, which include insulin resistance, hypertriglyceridemia, low high-density lipoprotein cholesterol concentration, central adiposity, and elevated blood pressure (BP) [1]–[4]. These risk factors covary in populations of varying age, gender and ethnicity [5]–[9]and, when combined in a single index, strongly predict incident cardiovascular disease and diabetes, disease course, and mortality [5], [7], [8], [10], [11]. Increasing evidence from the fields of genomics and metabolomics also identifies common pathways that contribute to multiple components of the metabolic syndrome [12]–[17]. Thus, considering the metabolic syndrome as a distinct entity has epidemiologic justification, although it remains unclear whether one or more pathways drive the observed covariation of these risk factors.

In contrast, the second metric, allostatic load, stems from a conceptual model of biological adaptations to cumulative environmental demands (allostasis) [18], [19]. It is hypothesized that recurrent activation of autonomic and neuroendocrine responses, elicited by exposure to stressful situations and varying in extent, frequency, or duration, engender a progressive dysregulation of multiple physiological systems. Proponents of the allostatic model distinguish between two components of allostasis, referred to as primary and secondary mediators. Prominent indicators of response to environmental challenges, such as the release of stress-related hormones (e.g., catecholamines, cortisol), a shift in sympatho-vagal balance, or the production of inflammatory cytokines, comprise the primary allostatic mediators. Secondary mediators reflect ensuing biological alterations that accumulate over time and confer risk for clinical disease. These include many of the same factors that define the metabolic syndrome. Allostatic theory posits a centrally-mediated orchestration of environmentally-induced biological adaptations, so that the index of allostatic burden (allostatic load) is typically constructed as a simple aggregate of primary and secondary mediators [20], [21]. Such summary scores have been shown to predict cognitive and physical functioning [21], [22], incident cardiovascular disease [20], [21] and all-cause mortality [20], [23]_ENREF_16. Further, the few studies that have examined the primary and secondary components of allostatic load separately suggest that primary mediators may predict all-cause mortality independently of variables related to the metabolic syndrome [20], [23]. Whether covariation among the various indicators contributing to allostatic load warrants their treatment as a unitary construct remains to be established, however, and in particular, the relation of primary allostatic mediators to secondary mediators (e.g., metabolic syndrome) requires elucidation.

Confirmatory factor analysis (CFA) is a hypothesis-driven statistical technique developed to determine whether associations among multiple variables conform to one or another hypothesized underlying structure [24]. CFA models are comprised of a series of simultaneous regression equations, and the fit of these equations to observed data can be tested using model fit statistics [24], [25]. Within these models, one or more first-order factors can be posited. Such factors could reflect, for instance, associations among several closely related (and hence, highly intercorrelated) variables within a *common* physiological system (e.g., systolic and diastolic blood pressure; central adiposity and body mass index). First-order factors may also covary to yield one or more second-order factors. Such second-order factors may be used to describe association across measures from *different* physiological systems (e.g., a single factor underlying both a first-order blood pressure factor, defined by systolic and diastolic blood pressure, and a first-order adiposity factor, defined by BMI and waist circumference).

CFA has shown prior utility in examining the mathematical structure underlying metabolic syndrome [26]–[32]. In applying CFA to the standard elements of the metabolic syndrome, for instance, first-order factors representing each of the four component systems (namely, insulin resistance, obesity, dyslipidemia and elevated BP) could be unified under a single second-order factor, labeled “metabolic syndrome”, and this hierarchical structure provided a good fit across different samples, and in men and women [29]–[32]. Evidence of a single common factor underlying interindividual variability in these several physiological systems provides additional biometric validation for the metabolic syndrome as a coherent entity.

To our knowledge, this statistical methodology has not been applied to measures associated with allostatic load. Good model fit with a single, common factor would provide novel, empirical evidence of allostatic load as a unitary construct. Further, CFA can be used to examine whether components of allostatic load continue to covary when the metabolic syndrome is conjointly modeled. Accordingly, we employ CFA in the present study to test: 1) whether a single common factor underlies variation in physiological systems associated with allostatic load (as available in the present study); and 2) whether allostatic load parameters continue to load on a single common factor if a second factor representing the metabolic syndrome is also modeled.

## Methods

### Participants

Data for the present study were derived from the University of Pittsburgh Adult Health and Behavior project, a registry of behavioral and biological measurements on Non-Hispanic Caucasian and African American individuals (30–54 years old) recruited via mass-mail solicitation from communities of southwestern Pennsylvania, USA (principally Allegheny County)[31], [33]–[36]. Exclusion criteria for entry into the parent study included a reported history of atherosclerotic cardiovascular disease, chronic kidney or liver disease, cancer treatment in the preceding year, neurological disorders, or psychotic illness. Other exclusions included pregnancy and the use of insulin, nitrates, glucocorticoid, antiarrhythmic, psychotropic, or prescription weight-loss medications. This study was approved by the University of Pittsburgh Institutional Board. Written informed consent was obtained in accordance with approved protocol guidelines of the University of Pittsburgh Institutional Review Board.

Additional exclusion criteria for the current analyses included use of antihypertensives, oral hypoglycemics, cholesterol-lowering medications, immunosuppressants, cold medications or antibiotics. Of the 1007 members of the parent project who met the above criteria, circulating interleukin-6 (IL-6) and C-reactive protein (CRP) concentrations were available for a subsample of 723 individuals. Of these, 73 individuals with IL-6 levels greater than 10 pg/ml or CRP levels greater than 10 mg/L, suggesting the presence of acute illness (e.g., common respiratory infections), and 5 individuals who were missing components of the metabolic syndrome were dropped, resulting in a final sample of 645 individuals.

### Measures

The measures included in the present analysis were systolic and diastolic blood pressure, body mass index (BMI) and waist circumference, fasting glucose, insulin, high-density lipoprotein cholesterol, triglycerides, plasma IL-6 and CRP, and paced and unpaced heart rate variability at rest. Participants were asked to fast overnight for 8 hours and avoid exercise for 12 hours and alcohol for 24 hours before coming into the laboratory in the morning to have blood drawn. At this visit, a nurse completed a medical history and medication use interview, obtained measurements of height and weight for the determination of BMI (kg/m^2^), took two manual BP measurements, obtained a measurement of waist circumference, and drew a 40-mL blood sample. BP measurements were made after the subject was seated for 20 minutes with the arm supported, using the appropriate cuff size for the subject’s arm circumference. Serum glucose, HDL-cholesterol, and triglyceride concentrations were measured by the Heinz Nutrition Laboratory, School of Public Health, University of Pittsburgh, which has met criteria for the Centers for Disease Control and Prevention – National Heart, Lung, and Blood Institute Standardization Program since 1982. Insulin concentration was measured in duplicate with a radioimmunoassay (Code-a-count; Diagnostic Products, Inc, Los Angeles, CA). IL-6 levels were determined in duplicate by high sensitivity quantitative sandwich enzyme immunoassay kit (R & D Systems, Minneapolis, MN) according to manufacturer’s directions. CRP was measured at the University of Vermont’s Laboratory of Clinical Biochemistry Research with the BNII nephelometer from Dade Behring utilizing a particle enhanced immunonephelometric assay. These methods were previously described in detail by Marsland and colleagues, 2010 [31].

To measure resting cardiac vagal activity, heart rate was recorded continuously using a 2-lead electrocardiogram (ECG) attached bilaterally to the wrists. While seated in a temperature and sound-controlled chamber and after a 10-minute rest period, two successive 5-minute, resting ECG recordings were obtained. During the first, patients were instructed to relax and breathe at a comfortable rate (unpaced breathing). During the second (paced breathing), participants were asked to breathe naturally in response to two auditory tones signaling them to inhale and exhale. Respiration was paced at a rate of 11 breaths/minute based on pilot observations that affirmed this is a comfortable rate for most people. Respiration was monitored throughout using a thoracic strain-gauge. ECG signals were digitized at a sampling rate of 1000 Hz (LabView acquisition software, National Instruments Corporation, Austin, Texas). Before calculating estimates of HRV, the digitalized ECG signals were examined and artifactual detections of R-wave occurrences were corrected. All procedures and analyses followed Task Force guidelines [37].

The sequential cardiac interbeat interval time series from the paced and unpaced resting baselines were assessed to determine the component frequencies using a point process analysis developed at the University of Amsterdam, PSPAT [38]. This program yields results similar to a Fourier decomposition, but does not assume a continuous underlying generator function. Conceptually, the analysis is consistent with the integral-pulse frequency-modulation approach used in recent modeling of the neural basis of HRV [39]. High frequency HRV (HFHRV) was defined as 0.15 Hz–0.39 Hz. The square root of the mean of the squares of successive normal-to-normal interval differences (RMSSD) was also calculated for the measurements obtained under paced and unpaced respiration.

CRP, insulin, glucose, triglycerides, RMSSD and HFHRV values were log normal (base _e_) transformed to better approximate a normal distribution. Reciprocal transformation was applied to normalize raw score distributions of the IL-6 values.

### Data Analysis

Confirmatory factor analysis was conducted based on Bentler and Weeks’ model [24] using the EQS program [25]. Tests of significance were set at.05 (two-tailed). The ratio of cases to variables was over 50∶1, and the ratio of cases to parameters was 16∶1. Both were sufficient for conducting CFA. A chi-square test was used to evaluate the congruency between the hypothesized model and empirical data, although it is well recognized that chi-square tests are sensitive to large sample size [40], [41]. As such, 3 other model fit indices were used: comparative fit index (CFI; 0.95 or above; indicative of good fit), average absolute standardized residuals (0.05 or less; indicative of good fit), and root mean square error of approximation (RMSEA; 0.05 or less; indicative of good fit) [25].

Age, sex and race were statistically adjusted in each analysis. As models including paced or unpaced respiration produced similar results and 20 participants were missing data for unpaced respiration, we present models with paced respiration in the manuscript. A model substituting unpaced respiration is presented in Figure S1. The first step was a confirmatory factor analysis of the metabolic syndrome components. Fasting insulin and glucose, BMI and waist circumference, fasting HDL-cholesterol and triglycerides, SBP and DBP were arranged by physiological system to load on four first-order factors, “insulin resistance”, “adiposity”, “dyslipidemia”, and “BP”. The model included a single second-order latent factor, hypothesized to underlie common variability among the four first-order factors, consistent with the conceptualization of the metabolic syndrome. We have published this model previously [31] but present it here as it forms the basis for the later models.

In the second model, we added an inflammation factor, defined by IL-6 and CRP, and a vagal tone factor, defined by HFHRV and RMSSD during paced respiration, to the metabolic syndrome model from above to determine whether all variables included in the conceptualization of allostatic load and available in this study were associated with a single second-order factor, which we label the “allostatic load” factor.

In the third model, we modeled two second-order factors and allowed the multiple first order factors to load on both an “allostatic load” factor, comprised of blood pressure, lipids, adiposity, insulin resistance, inflammation and HRV factors, and a “metabolic syndrome” factor, comprised of blood pressure, lipids, adiposity and insulin resistance factors. This model tests whether the data are consistent with the presence of a factor representing allostatic load while conjointly modeling a factor representing metabolic syndrome, as previously defined. Good model fit of this two factor model would indicate distinct covariance between metabolic syndrome and allostatic load variables.

As systemic inflammation is strongly associated with the metabolic syndrome and, indeed, has been proposed as a component of the syndrome [31], in the fourth model we examined whether any residual correlation independent of the metabolic syndrome may be attributed solely to inflammation. Similarly, in a fifth model, we examined whether any residual correlation independent of the metabolic syndrome may be attributed solely to vagal tone.

## Results

### Sample Characteristics

Demographic characteristics of the sample and descriptive statistics of the metabolic, vagal and inflammatory variables are displayed in Table 1. Participants were on average 45 years of age, 82% non-Hispanic white, 52% female and 16% current smokers. The sample was on average in the overweight range with average serum lipids and blood pressures in the normal range.

**Table 1:** Descriptive statistics of participants’ demographic and biomedical characteristics.

	Mean or %	SD
Age	44.65	6.55
Sex (male/female)	48%/52%	
Race (European-Americans/African Americans)	82%/18%	
Education (years)	15.60	2.74
Current smokers	15.8%	
Insulin (uU/ml)	12.79	6.53
Glucose (mg/dl)	95.81	16.68
Body mass index (kg/m^2^)	27.16	5.38
Waist circumference (inches)	35.85	5.87
High density lipoprotein cholesterol (mg/dl)	54.05	14.19
Triglycerides (mg/dl)	120.55	86.93
Systolic blood pressure (mmHg)	116.35	13.27
Diastolic blood pressure (mmHg)	78.50	9.43
Interleukin-6 (pg/ml)	1.79	1.68
C-reactive protein (mg/L)	1.65	1.79
Paced RSSMD	36.96	29.83
Unpaced RSSMD	36.05	23.17
Paced high frequency band	63484.76	3911.84
Unpaced high frequency band	33016.56	1994.70

### Model 1: Do Components of the Metabolic Syndrome Load on a Single Common Factor?

**Figure 1:**
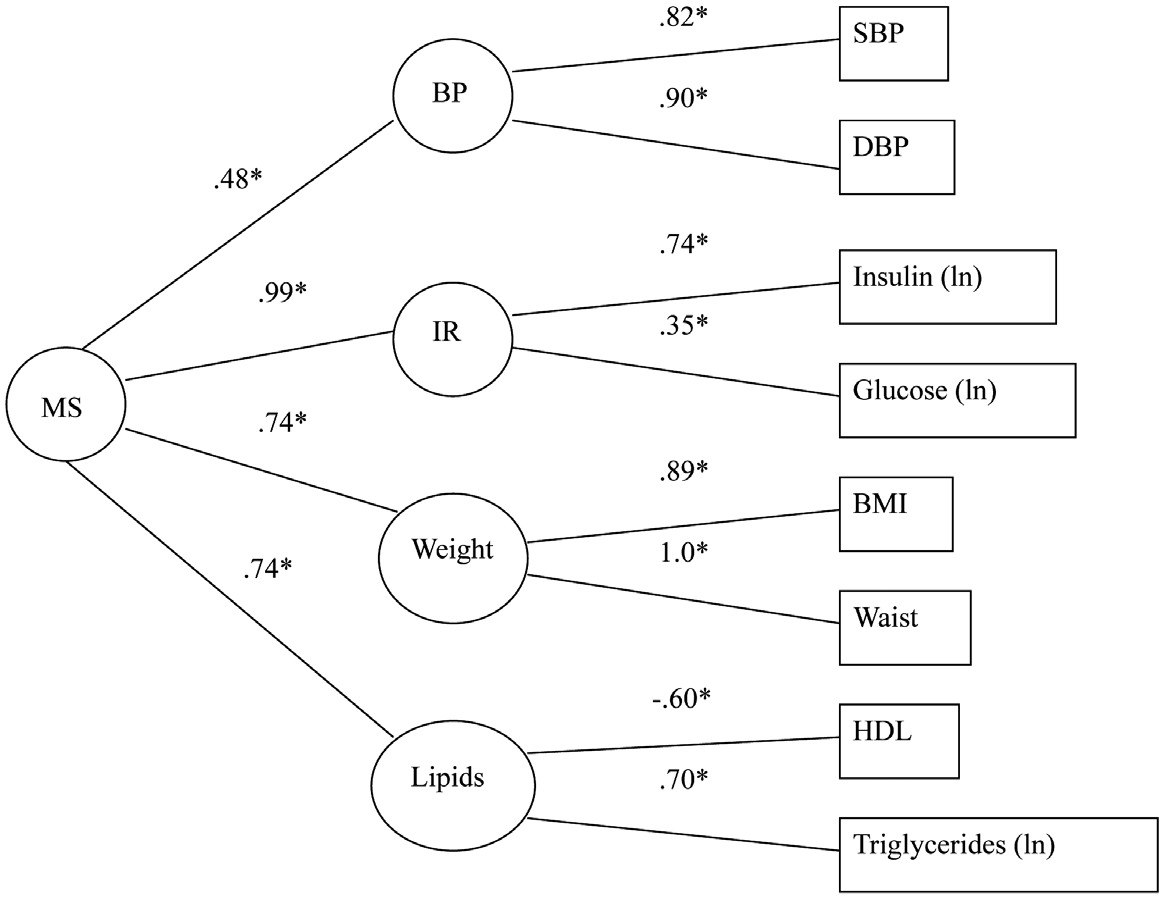
Model 1: Factor structure of the metabolic syndrome. Age, sex and race covaried; relevant medications excluded. MS – Second-order metabolic syndrome factor; IR – insulin resistance factor; boxes represent indicator variables and circles reflect latent factors. χ^2^ = 57.51, df = 12, p<0.001, N = 645; CFI = .98, average absolute standardized residuals = .02, RMSEA = .08.

As presented previously [31], CFA of the metabolic syndrome provided a reasonably good fit to the data in this sample (Figure 1). The CFI of the model was 0.98, with the average absolute standardized residual = 0.02 and RMSEA = 0.08. Although the statistically significant chi-square test (χ^2^ = 57.51, df = 12, N = 645, p<0.01) indicated some difference between the estimated and observed variance-covariance matrices; it is well known that χ^2^ statistics are sensitive to large sample sizes and it is recommended to employ multiple fit statistics in this context [40], [41]. The measured variables tended to load strongly on their respective factors, with the potential exception of the loading of glucose on the insulin resistance factor. Each of the subfactors loaded significantly on the second-order factor. The good fit for the metabolic syndrome model provides a strong basis from which to evaluate whether the factor structure underlying other potentially correlated variables is independent of variance attributable to the metabolic syndrome.

### Model 2: Do Allostatic Load Components Load on a Single Common Factor?

**Figure 2:**
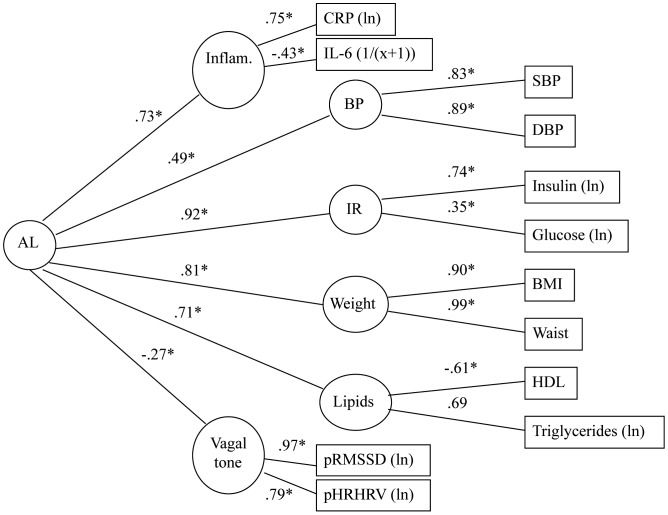
Model 2: Single second-order factor model: common factor underlying allostatic load parameters. Age, sex and race covaried; relevant medications excluded. AL – Second-order allostatic load factor; IR – insulin resistance factor; boxes represent indicator variables and circles reflect latent factors. χ^2^ = 145.05, df = 42, p<0.001, N = 645; CFI = .97, average absolute standardized residuals = .03, RMSEA = .06.

In the next model, we determined whether an inflammatory factor (defined by IL-6 and CRP), a vagal factor (defined by HFHRV and RMSSD under paced respiration at rest), and “insulin resistance”, “adiposity”, “dyslipidemia”, and “BP” factors (as defined above) could all be represented with a single common underlying factor (Figure 2). The CFI of the model was 0.97, with the average absolute standardized residual = 0.03 and RMSEA = 0.06, indicative of good model fit. Again, although the statistically significant chi-square test (χ^2^ = 145.05, df = 42, N = 645; p<0.001) indicated some difference between the estimated and observed variance-covariance matrices, this was likely due to large sample size. IL-6 and CRP showed a moderate to strong association with the “inflammation” factor. The path coefficient for the association between inflammation and the common, second-order factor was substantial. For the vagal measures, HFHRV and RMSSD loaded strongly on a “vagal tone” factor. This factor, in turn, was moderately associated with the second-order factor. The negative path coefficient indicated that higher levels of the metabolic and inflammatory parameters were associated with lower vagal tone. The model including HFHRV and RMSSD during unpaced respiration produced similar results and is presented in Figure S1. Overall, these results indicated that a single common factor underlies variation in allostatic load components, as available in the present study.

### Model 3: Do Allostatic Load Parameters Load on a Single Common Factor Independent of the Metabolic Syndrome Factor?

**Figure 3:**
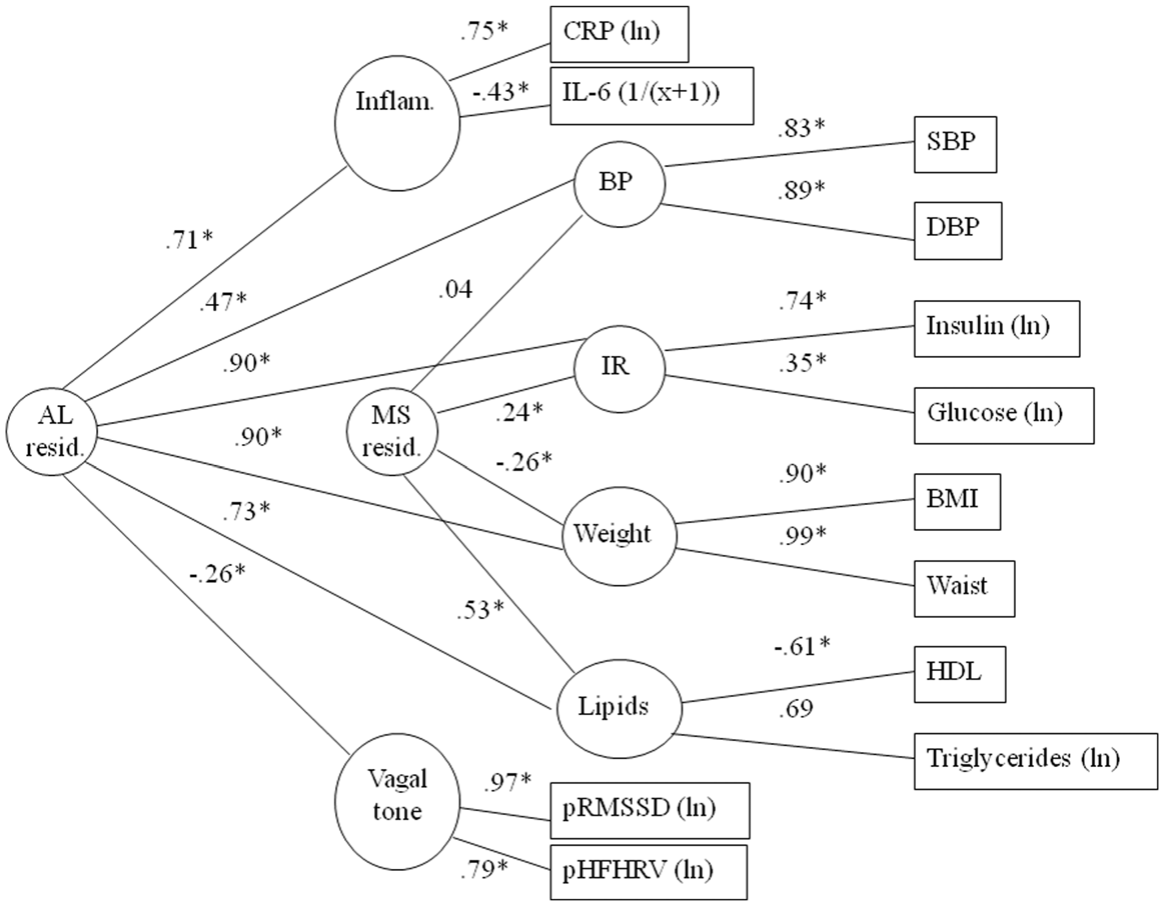
Model 3: Two second-order factor model: allostatic load and metabolic syndrome factors. Age, sex and race covaried; relevant medications excluded. MS resid.: Second-order metabolic syndrome factor with allostatic load parameters simulataneously modeled; AL resid.: Second-order allostatic load factor with metabolic syndrome pathways simultaneously modeled; IR – insulin resistance factor; boxes represent indicator variables and circles reflect latent factors. χ^2^ = 125.00, df = 38, p<0.001, N = 645; CFI = .97, average absolute standardized residuals = .02, RMSEA = .06. Δχ^2^ (4) = 20.05, p<0.01.

In the third model, we aimed to determine whether there was evidence for covariation among the allostatic load parameters when a metabolic syndrome factor was conjointly modeled (Figure 3). Overall, the model with two second-order factors provided a good fit to the data with CFI, average absolute standardized residual and RMSEA within acceptable ranges (χ^2^ = 125.00, df = 38, p<0.001, N = 645; CFI = .97, average absolute standardized residuals = .02, RMSEA = .06). This result provides evidence for distinct factors representing allostatic load and metabolic syndrome parameters.

As Model 2 is nested within Model 3, we can use the change in chi-square between the models to determine which model provides a better fit to the data. The improved fit for Model 3 relative to Model 2 (Δχ^2^ (4) = 20.05, p<0.01), suggests that a model with two second-order factors, representing both allostatic load and metabolic syndrome, is significantly more consistent with the observed data that a one second-order factor model. This provides further evidence for a factor representing allostatic load, independent of a metabolic syndrome factor, but also raises the possibility that two constructs underlie allostatic load as represented by this set of indicators.

We note that when two second-order factors are conjointly modeled, the allostatic load second-order factor is represented by all putative components. Although the metabolic syndrome residual factor has components of cardiometabolic risk, unexpectedly, blood pressure does not contribute meaningfully and the weight factor has an anomalous loading. This occurs despite factor loading consistent with theory when the metabolic syndrome is modeled independently (Figure 1). This result likely suggests that the allostatic load and metabolic syndrome constructs, as defined in this paper, are strongly interrelated and, despite evidence of independent constructs from the overall change in model fit, individual path co-efficients may be estimated with some uncertainty.

### Supplementary Analyses: Is the “Allostatic Load” Factor Solely Attributable to Inflammation or Vagal Tone?

Model 3 shows that allostatic load is not just synonymous with the metabolic syndrome, but is the residual covariance of risk indicators attributable only to the inclusion of inflammation, or alternatively, only to the inclusion of vagal tone? In Model 4 (Figure S2), we built upon Model 3 to determine whether the second-order factor representing allostatic load parameters might be attributable to inflammation alone. Forcing the second-order factor to reflect inflammation only resulted in acceptable model fit statistics (CFI = .97, average absolute standardized residuals = .03, RMSEA = .06) but a significant decrement in model fit relative to Model 3 (Δχ^2^ (1) = 16.44, p<0.001). This indicated that a model in which the second-order factor was defined by both inflammation and vagal tone first-order factors fit the data better than a second-order factor defined by inflammation alone. Thus, inflammation did not fully account for additional covariation between allostatic load parameters independent of the covariation among metabolic parameters.

In Model 5, we determined whether covariation between allostatic load parameters, independent of the metabolic syndrome, is solely attributable to vagal tone. Defining the second-order factor by vagal tone alone resulted in a significant decrement in model fit (Δχ^2^ (1) = 182.52, p<0.001) and also yielded unacceptable overall model fit statistics (CFI = .92, average absolute standardized residuals = .06, RMSEA = .10). As such, factor loadings for Model 5 are not presented. The significant decrement in model fit indicated that a model in which the second-order factor was defined by both inflammation and vagal tone first-order factors fit the data better than a second-order factor defined by vagal tone alone. Like inflammation, then, vagal tone did not fully account for the residual covariation among allostatic load indicators (when adjusted for a second-order factor reflecting the metabolic syndrome), indicating that optimal structure is attained by inclusion of both vagal tone and inflammatory markers.

## Discussion

The present study sought to determine: a) whether the pattern of covariation among various indicators of allostatic load merits interpretation of allostatic load as a unitary construct; and b) the relation of such a construct to characteristics of the metabolic syndrome. Our results support a single, second-order factor underlying the allostatic load components available in this study (metabolic, inflammatory and vagal measures). Further, the common, latent factor reflecting covariation among allostatic load components persisted when a second latent factor representing metabolic syndrome facets was conjointly modeled. Thus, this study demonstrates that allostatic load components share common variance, as hypothesized [18], [19], and that this common variance does not simply reflect correlation of allostatic load components with the metabolic syndrome. Unlike most prior studies, we utilized CFA to define allostatic load and metabolic syndrome. This measurement model approach garners several analytic advantages, including the use of the continuous distributions of the measured parameters and lack of reliance on thresholds, which may be sample-specific or lack validation. Further, the magnitude of factor loadings is determined empirically and does not rely on an assumption that all component measures contribute with equal weighting. Most notably, CFA can also provide an empirical test of whether a hypothesized model structure is consistent with the patterns of association that are obtained in observed data. _ENREF_37 Future applications of CFA in relation to the metric of allostatic load could include the use of allostatic load factors to predict age-related outcomes (e.g., cognitive and physical function, cardiovascular disease) and development of scoring systems of potential clinical utility [42].

It is notable that although one second-order factor unifying the first-order factors of blood pressure, dyslipidemia, insulin resistance, adiposity, inflammation and vagal tone provided a good fit to the data, the model incorporating two second-order factors – one reflecting all of the allostatic load components and the second reflecting metabolic syndrome components only – did provide a significantly improved fit. This result indicates that residual variance in allostatic load parameters form a common factor even when adjusted for the metabolic syndrome. Nonetheless, it also raises the possibility that two constructs may underlie variability in measures commonly used to index allostatic load, at least for the measures available in this study. It is plausible that one factor may represent primary mediators, such as stress-related hormones, sympatho-vagal balance and inflammatory cytokines, whereas the second factor may reflect secondary mediators that typically emerge over time, such as progressive changes in cardiovascular and metabolic function. Further validation studies, optimally including additional measures, both of primary mediators not available here (e.g., cortisol, epinephrine and norepinephrine) and secondary mediators not subsumed by the metabolic syndrome (e.g., peak expiratory flow [43]), will help to clarify whether a model incorporating two second-order factors holds as well across a broader array of physiological parameters.

We note, however, that the measures of allostatic load available in this study could be construed as an extended cardiometabolic risk profile, and not a distinct construct per se. Systemic inflammation, in particular, is closely related to adipose tissue health and may play a primary pathogenic role in the metabolic syndrome [44]. Further, the inverse association between vagal tone and markers of systemic inflammation observed in this study could be consistent with the “cholinergic anti-inflammatory pathway” [45], [46], which describes bi-directional communication between vagus nerve fibers and inflammatory mediators observed in animal models [46]–[48] and epidemiologic studies [49]–[52]. This again underscores the need for additional studies with greater representation of the multiple physiological systems currently hypothesized to represent allostatic load, to test whether allostatic load persists independent of a broad definition of cardiometabolic risk.

Evidence from genomics and metabolomics is beginning to characterize genetic pleitropy and common biology underlying metabolic syndrome [12], [13], [15]–[17]. The cluster of fatty acid desaturase genes 1–3 (FADS1-3), which has been associated with lipids [53], glucose and insulin [54], [55] and resting heart rate [56] in genome-wide association studies, is now an established predictor or polyunsaturated fatty acids and their ratios in metabolomic studies [17], [57], [58]. Glucokinase (hexokinase 4) regulator (GCKR), a pleiotropic risk locus associated with fasting glucose, insulin and triglyceride levels [15], is strongly associated with mannose, a derivative of glucose involved in glycosylation, and mannose:glucose ratio [17]. Adiponectin, a marker of adipose tissue health, predicts all facets of the metabolic syndrome as well as systemic inflammation [12], [14].

It is hypothesized that chronic stress and associated overactivation of stress hormones and pro-inflammatory cytokines affect a range of cellular and metabolic activities in allostatic load [18], [19], [43]. Genomic and metabolomic studies could establish overlapping and distinct pathways contributing to metabolic syndrome and allostatic load. A greater understanding of the structure of these constructs through CFA modeling provides an important basis for such research.

The present findings should be interpreted in the context of a number of limitations. First, our study is cross-sectional, which precludes causal interpretation of relationships among factors or indication of stability or change in model structure over time. In addition, our study was limited to European- and African-Americans, potentially limiting generalizability. Finally, the conceptual model (allostasis) from which allostatic load is derived posits several primary mediators, some of which (e.g., cortisol, catecholamines) were not available in this data set.

Despite these shortcomings, our findings demonstrate that peripheral markers of systemic inflammation, vagal tone and components of the metabolic syndrome load on a common latent factor. In addition, these measures continued to load on this common, latent factor when conjointly modeled with a second latent factor reflecting facets of the metabolic syndrome. These observations provide novel support for the common variance of diverse physiological systems postulated in the construct of allostatic load.

## Supporting Information

Figure S1 Single second-order factor model: common factor underlying allostatic load parameters including vagal tone during unpaced respiration. Age, sex and race were covaried; relevant medications excluded. AL – Second-order allostatic load factor; IR – insulin resistance factor; boxes represent indicator variables and circles reflect latent factors. χ^2^ = 161.28, df = 42, p<0.001, N = 625; CFI = .96, average absolute standardized residuals = .03, RMSEA = .07.
(TIF)

Figure S2 Model 4: Two second-order factor model: residual inflammation and metabolic syndrome factors. Age, sex and race were covaried and relevant medications excluded; relevant medications excluded. MS resid. – Second-order metabolic syndrome factor simultaneously modeled with an inflammation factor; IR – insulin resistance factor; boxes represent indicator variables and circles reflect latent factors. χ^2^ = 141.44, df = 39, p<0.001, N = 645; CFI = .97, average absolute standardized residuals = .03, RMSEA = .06. Δχ^2^(1) = 16.44, p<0.001.
(TIF)
