# Supplementary figures and images for: Factor Structure Underlying Components of Allostatic Load
Source: PLoS One. 2012 Oct 24;7(10):e47246. doi: 10.1371/journal.pone.0047246 (PMC3480389; doi:10.1371/journal.pone.0047246)

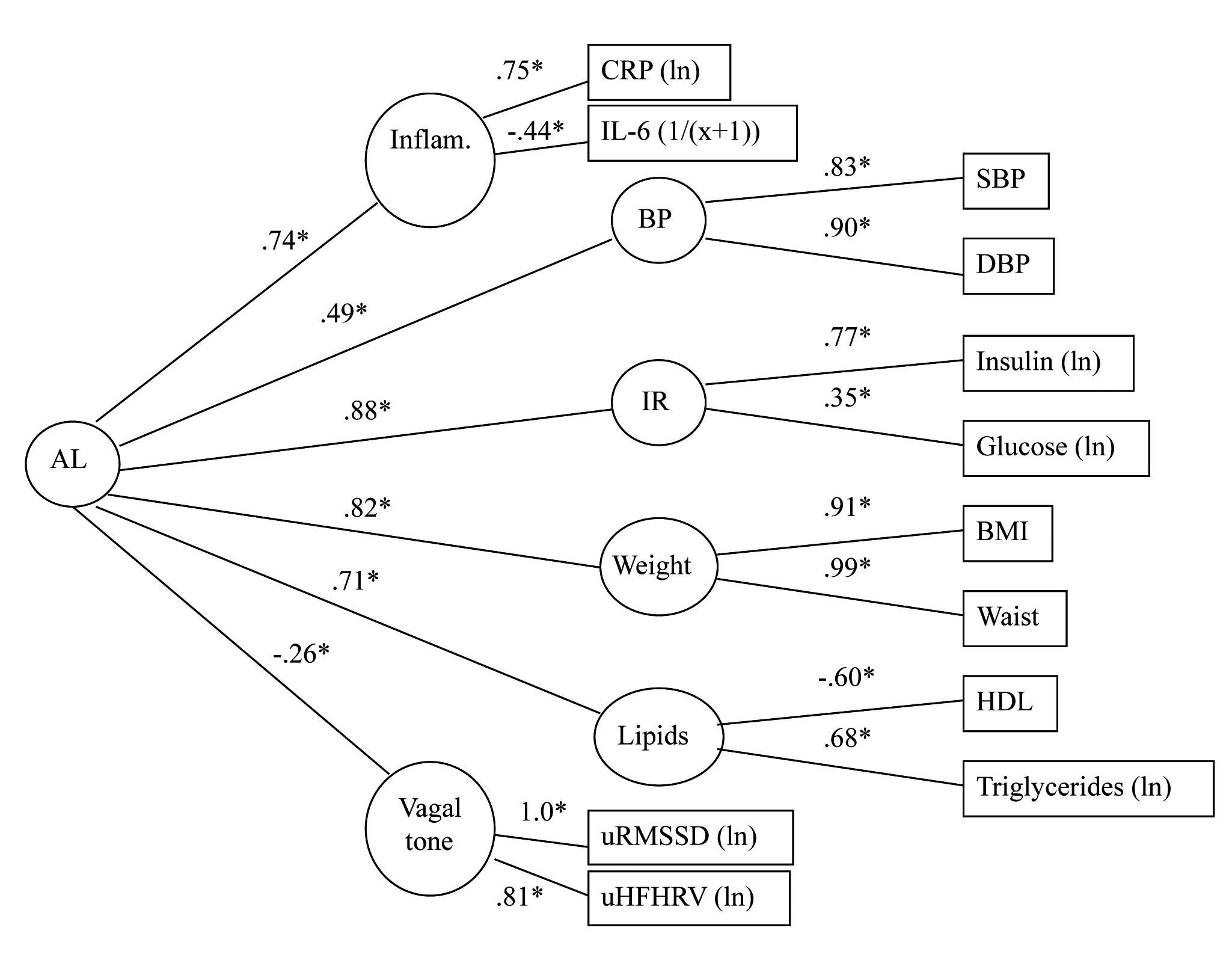

Supplement: Figure S1 — Single second-order factor model: common factor underlying allostatic load parameters including vagal tone during unpaced respiration. Age, sex and race were covaried; relevant medications excluded. AL – Second-order allostatic load factor; IR – insulin resistance factor; boxes represent indicator variables and circles reflect latent factors. χ2 = 161.28, df = 42, p<0.001, N = 625; CFI = .96, average absolute standardized residuals = .03, RMSEA = .07. (TIF) [file pone.0047246.s001.tif]
